# Seductive details hamper learning even when they do not disrupt

**DOI:** 10.1007/s11251-023-09632-w

**Published:** 2023-05-12

**Authors:** Anna Kienitz, Marie-Christin Krebs, Alexander Eitel

**Affiliations:** Department of Educational Psychology, Justus-Liebig-Universität Gießen, Otto-Behaghel-Str.10D, 35390 Gießen, Germany

**Keywords:** Multimedia learning, Learning with multiple representations, Seductive details, Decorative images, Relevance instruction, Cognitive load

## Abstract

Previous research often revealed detrimental effects of seductive details on learning with multimedia instruction, but there are mixed findings regarding how to best explain these detrimental effects. We investigated whether the detrimental effects of seductive details are mainly mediated by the cognitive processes of diversion (deeper processing of seductive details rather than pertinent content) or disruption (unsuccessful attempts to integrate seductive details with pertinent content) by assessing the effects of instructional prompts. In an online learning experiment, participants (*N* = 247) learned either without seductive details (control condition) or with seductive details in one of three conditions: Participants received either a prompt informing them about the irrelevance of seductive details (irrelevance-prompt), a prompt to process seductive details and pertinent content separately (separation-prompt), or no prompt within their task instruction. We assessed recall and transfer of knowledge as dependent variables. Supporting the diversion hypothesis, participants in the no-prompt condition regarded seductive details as more relevant and consequently spent more time processing them compared to participants in the irrelevance-prompt condition, which negatively influenced their recall performance. Against the disruption hypothesis, participants in the no-prompt condition reported lower integration avoidance between seductive details and pertinent content compared to participants in the separation-prompt condition, but this led to better rather than worse transfer performance. Our results thus suggest diversion, and not disruption, to be the main process driving the seductive details effect. Reducing the details’ diverting potential seems a good way to deal with seductive details in instruction.

## Seductive details hamper learning even when they do not disrupt

With the increasing relevance of self-directed online learning since the COVID-19 pandemic, the question of how to make learning more engaging for students is increasingly pressing. One way to potentially increase learning engagement is to add eye-catching and interesting details (e.g., comics, fun-facts) to online learning materials. While these kinds of details try to make e-book pages look more appealing, they also pose a threat to learning when they are irrelevant for grasping the main topic. In research, such interesting but irrelevant parts of learning material are labelled *seductive details* and had detrimental effects on learning (Garner et al., 1989). However, it is yet an open question what process mainly drives the negative effects of seductive details on learning performance. Consequently, the question remains how to deal effectively with seductive details in instruction. The present research wants to provide an answer to both questions by testing how different types of instructional prompts affect learning with seductive details.

### The seductive details effect

Empirical studies often found that enriching an instruction with interesting but irrelevant details (i.e., seductive details) led to worse scores for recall and comprehension concerning the relevant instructional contents (e.g., Lehman et al., 2007; Eitel et al., 2019). For example, adding pictures with short anecdotes about the effects of lightning strikes to an instruction about the formation of lightning led to worse score for recall and comprehension of lightning formation (Harp & Mayer, 1998). This effect is known as the seductive details effect (Garner et al., 1989). The effect is of small to moderate size when looking at the aggregated research findings from the past 35 years (see Rey 2012; Sundararajan & Adesope, 2020, for meta-analyses).

The seductive details effect is commonly explained by referring to the cognitive load theory (CLT; Sweller et al., 2011). According to CLT, students experience different types of cognitive load while learning. Intrinsic cognitive load (ICL) occurs when making sense out of learning material and, thus, is integral to the processing and understanding of the new content. A higher experienced ICL can, for example, result when learning material requires many inferences and connections within itself for understanding. Extraneous cognitive load (ECL) is additional load that is unnecessary for understanding the contents, can cause cognitive overload and thus may hinder learning. ECL reflects learners’ efforts to deal with the design of (suboptimal) instruction. For example, ECL can be increased by adding interesting yet irrelevant contents to learning material (seductive details). Accordingly, increased ECL mediated the seductive details effect in previous research (e.g., Eitel et al., 2019).

Other explanations of the seductive details effect can be derived from the cognitive theory of multimedia learning (CTML; Mayer 2014). According to CTML, the process of meaningful learning involves three steps: Learners select relevant information for further processing, organize the selected information into pictorial and verbal mental models, and integrate organized information from the two mental models into a coherent mental representation together with prior knowledge. Presenting seductive details can have detrimental effects by interfering with each of the three processing steps (Harp & Mayer, 1998): Seductive details may (1) distract from selecting relevant information (i.e. distraction explanation), (2) disrupt coherence formation when organizing mental models (i.e. disruption explanation)^1^, and (3) divert by activating and integrating inadequate prior knowledge to form mental models about irrelevant contents (i.e. diversion explanation). It is not yet fully resolved which one of the three processes is the most detrimental to learning, which is in the focus of this research. Previous research yields a different degree of support for each of the processes.

First, there is some empirical support for *distraction* being detrimental for learning, because seductive details effects were stronger in studies with limited compared to unlimited study times (Rey, 2012). This finding supports the distraction explanation, because distracting from selecting relevant information should be more detrimental when there is not much time to select information overall. In addition, findings from eye-tracking studies tentatively support distraction by showing that seductive details reduced attention on relevant pictures (Park et al., 2015). This is important because the degree to which learners pay attention to relevant pictures, in turn, affect retention and comprehension performance (Eitel, 2016; Korbach et al., 2016).

Second, there is some indirect support for *disruption* to being detrimental to learning. Lehman et al. (2007) showed that seductive details hampered comprehension and slowed down reading times for passages that directly followed the seductive details – possibly reflecting coherence formation problems due to seductive details. This is consistent with findings of Wade et al. (1993). In interviews conducted during the study, learners stated that they had read seductive details slower while trying to integrate them with the remaining information.

Third, most empirical support points towards *diversion* being the most important process behind the seductive details effect. Harp and Mayer (1998) found seductive details being detrimental to learning when they were presented early but not late in the instruction suggesting that presenting seductive details early primed inadequate prior knowledge and led to deeper processing of irrelevant details at the expense of pertinent content. In a similar vein, Eitel et al. (2019) and Bender et al. (2021a) found that seductive details hampered learning only when students (mistakenly) considered them relevant for learning, which presumably led to increased prior knowledge activation and deeper processing of seductive details. Similar assumptions can be made based on the construction-integration model (Kintsch, 1998). After constructing propositional representations of the text (text base), learners integrate this mental representation with their prior knowledge into a more accurate representation of the text (situation model). When seductive details are present, learners could falsely integrate prior knowledge related to the seductive details, instead of pertinent contents, into the situation model. Accordingly, Chang and Choi (2014) demonstrated a negative link between gaze duration on seductive details and recall performance. A longer gaze duration might reflect deeper processing of seductive details by activating and integrating prior knowledge about the seductive details (i.e., diversion). Results from Bender et al. (2021) confirm this argumentation. Bender and colleagues (2021b) had students think aloud while inspecting their own eye movements from previously learning with (or without) seductive details. Students themselves reported more often that they felt diverted, rather than just distracted or disrupted, by the seductive details. The more students reported diversion, the worse their performance.

To sum up, most empirical evidence points towards diversion being the most relevant process that drives the negative effects of seductive details on learning performance. However, it is hardly possible to sharply distinguish distraction from the processes of diversion and disruption. Learners first have to allocate attention to the seductive information (distraction) in order for it to be processed instead of pertinent content (diversion) or to disrupt the formation of coherent mental models (disruption). We thus consider distraction being a necessary pre-condition for the diversion and the disruption explanation in our design.

### Is it just diversion that drives the seductive details effect?

Even though previous research points towards diversion mainly driving the seductive details effect (e.g., Bender et al., 2021b; Harp & Mayer 1998), it is still unclear whether it is just diversion that causally drives the effect, or whether disruption nevertheless plays an important role for the seductive details effect. In other words, it is still an open question whether seductive details hamper learning even if they do *not* disrupt. Previous research gathered process data (eye gaze, reading times, think aloud protocols; e.g., Bender et al., 2021b; Lehman et al., 2007) of students learning with or without seductive details to obtain evidence in favor of diversion (distraction, or disruption) but not to obtain evidence against one of the other two processes. Both types of evidence are important to focus the breadth of potential explanations of the seductive details effect on the most relevant one(s) to potentially derive tailored recommendations on how to deal with seductive details in instruction. Here we try to find both types of evidence by designing prompts that make diversion and/or disruption very (un-)likely to occur, and then observe whether there is still a seductive details effect.

Diversion means that students build mental models around irrelevant contents (included in seductive details) rather than around the pertinent content (Harp & Mayer, 1998), resulting in a mental model that includes mainly irrelevant content. Diversion is stronger when students erroneously consider the irrelevant content as relevant and consequently construct mental models around them. Accordingly, empirical research found that the perceived relevance of seductive details moderated the seductive details effect (Bender et al., 2021; Eitel et al., 2019). In this previous research, negative effects of seductive details (also on ECL) disappeared as soon as students received a prompt about the details’ being irrelevant for the instructional goal (the posttest). Thus, on the one hand, the results by Bender et al. (2021b) and Eitel et al. (2019) suggest that (preventing from) diversion may mainly underlie the seductive details effect. Receiving the irrelevance prompt reduced the perceived relevance of seductive details, leading to the construction of mental models around pertinent content rather than irrelevant contents. This then fostered learning performance.

On the other hand, disruption might have also played a role in their studies. Receiving the irrelevance prompt may have prevented students from unsuccessfully trying to organize one coherent mental model around both pertinent contents and seductive details. Trying to establish coherence between pertinent contents and seductive details, and failing to do so, mainly drives the seductive details effect according to the disruption explanation (Harp & Mayer, 1998). According to disruption, students who try to establish coherence between pertinent content and seductive details should show a seductive details effect. In consequence, students who do *not* try to establish coherence, because they know that, for instance, the seductive details include information about a separate topic that does not need to be connected to the pertinent contents, should thus not show a seductive details effect. Receiving a prompt about seductive details referring to a separate topic, according to the disruption explanation, should prevent from the seductive details effect by avoiding (unsuccessful) integration attempts. In this research, we investigate whether this holds true empirically.

### The current study and hypotheses

The current study tests the diversion and the disruption explanation using different types of prompts. More precisely, participants in one condition were informed about the irrelevance of seductive details, potentially leading to reduced diversion. In another condition participants were instructed to process seductive details and main ideas separately, potentially leading to reduced disruption. Overall, the experimental design comprised (1) a control condition without seductive details, and three more conditions with seductive details. In the conditions with seductive details, participants received either (2) a prompt about the seductive details being irrelevant, or (3) a prompt about the seductive details referring to a separate topic, or (4) no prompt. For this design, we have the following hypotheses:

1. *Seductive details hypothesis*: Aligning with the reported literature (e.g., Sundararajan & Adesope 2020) we expect to observe differences in learning success between the control condition (1) and the condition with seductive details without prompts (4). Participants in the control group should outperform participants learning with seductive details in recall as well as transfer performance.
2. *Diversion hypothesis*: Following the diversion explanation, seductive details should hamper learning especially when they are perceived as relevant (Eitel et al., 2019). Therefore, students who receive a prompt about the details’ irrelevance (2) should outperform students who do not receive such prompt (4), and should perform as well as students in the control condition without seductive details (1), concerning recall and transfer performance. Moreover, we expect these effects on recall and transfer to being driven by a two-step mediation effect. Receiving a prompt about the details’ irrelevance should reduce the perceived relevance of seductive details, increase the relative learning time spent on main ideas versus seductive details, and thus foster recall and transfer compared to not receiving such a prompt.
3. *Disruption hypothesis*: Following the disruption explanation, students should underperform when they (unsuccessfully) try to establish coherence between seductive details and pertinent contents of the instruction. Therefore, students who receive a prompt about the seductive details referring to a separate topic (3) should have better recall and transfer scores than students not receiving such a prompt (4). This effect should be mediated by the self-reported avoidance of integrating seductive details and relevant material.
4. *Cognitive load hypothesis*: In line with findings from Eitel et al. (2019), we expected ECL to mediate the detrimental effects of seductive details. Students who learn with seductive details and receive no prompt (4) should experience higher ECL than those receiving a prompt about the details’ irrelevance (2) and the control group (1), and thus show poorer learning performance regarding recall and transfer.

## Method

### Participants and design

Initially, 257 participants completed the online learning experiment. Nine participants were excluded from our analyses because they had stated the use of external resources for the learning examination or that they had not participated faithfully. Accordingly, a final sample of *N* = 248 participants (199 women, 49 men, *M*_age_ = 23.39, *SD* = 5.88) remained. 82 participants were undergraduates in the field of psychology, 159 participants were undergraduates in other fields of study and 7 participants had completed vocational training. An a priori power-analysis via G*Power 3.1 design (Faul et al., 2007, 2009) for an assumed medium effect size (alpha = 0.05; power = 0.9, *f* = 0.25; derived from prior studies by Rey 2012; Sundararajan & Adesope, 2020) resulted in a recommended sample size of *N* = 232. Participants were recruited via social media platforms and a voluntary student data base of two german universities. Participants either received course credit (1 h) or had the chance to win one of 30 Amazon gift cards (10€) as compensation. Participants were randomly assigned to one of four experimental conditions: The control condition (no seductive details; *n* = 60), the irrelevance-prompt condition (material including seductive details, prompt about irrelevance of seductive details; *n* = 66), the separation-prompt condition (material including seductive details, prompt about separate processing of seductive details; *n* = 64), and the no-prompt condition (learned with seductive details, no instructional prompts; *n* = 58). The reported study was approved by the local ethics board (LEK FB06 2020-0037).

### Materials and experimental manipulations

In all conditions, a German translation of learning materials by Harp and Mayer (1998) and Mayer and Moreno (1998) was used (cf. Eitel et al., 2019). The material contained text and illustrations depicting causal processes involved in lightning formation. The text comprised 537 words and four explanatory illustrations. The illustrations were monochromatic schematic line drawings illustrating single steps of lightning formation. Participants were shown the learning material on a single page. Text and illustrations concerning lightning formation will be referred to as base passage in the following text.

In three conditions, the materials additionally included five seductive details. They were each presented as coloured decorative photographs with an associated description. The seductive details comprised 229 words and five colored photographs. Instead of dealing with lightning *formation* they described *consequences* of lightning strikes (e.g. a photograph showing a large beach with the description “swimmers are sitting ducks for lightning ”). Seductive details thus were related to the learning topic (lightning) and interesting but irrelevant for understanding (Garner et al., 1989). Hence, they match the definition of *instructional irrelevance* (Alexander, 2019).

**Fig. 1:**
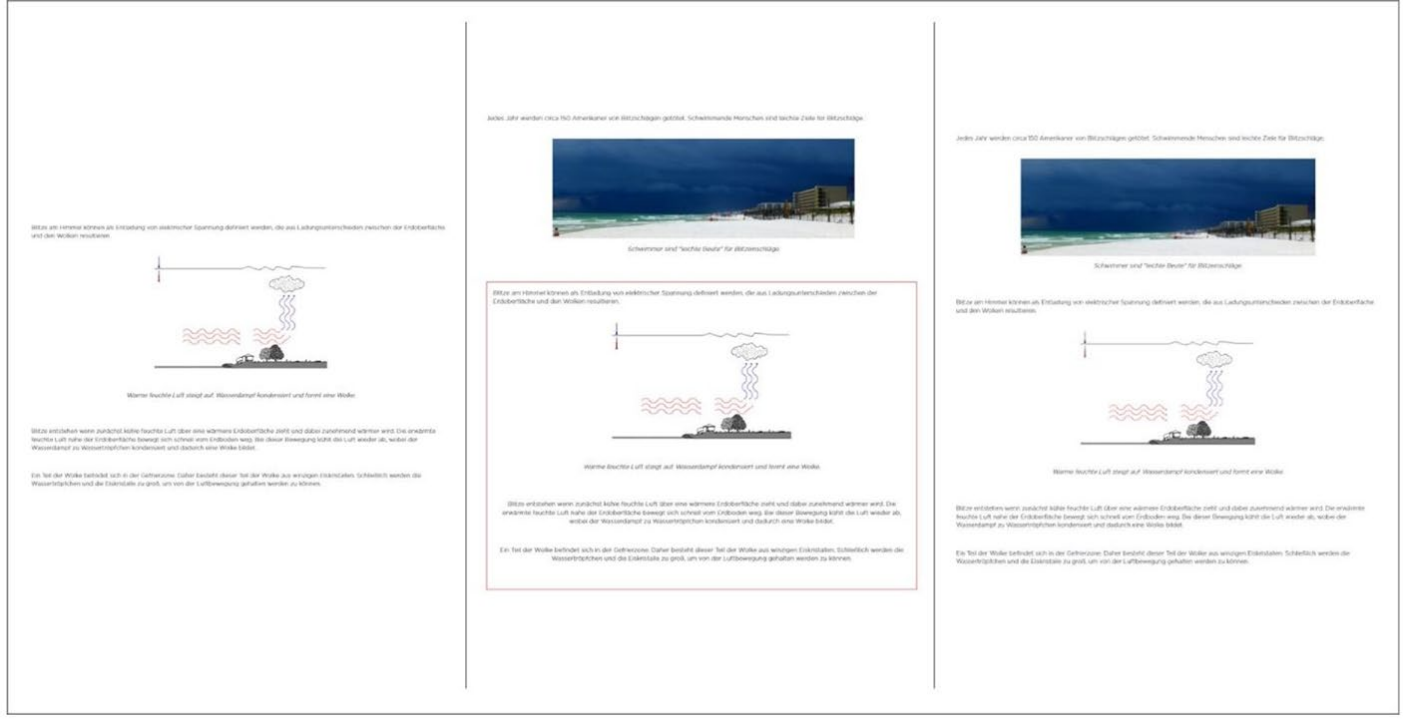
Excerpts from the learning materials used across the experimental conditions (left = control condition | center = separation-prompt condition & irrelevance-prompt condition | right = no-prompt condition)

In two of the four conditions, the irrelevance-prompt condition and the separation-prompt condition, the instruction on the front page contained additional prompts for the processing of the learning content. In the irrelevance-prompt condition, participants received the following prompt before the learning phase: “Please note that there will be several labelled photographs of the consequences of lightning strikes in the learning material. The information on these photographs is not relevant to the learning objective and will not be tested later. The only information relevant to the learning objective is information about the formation of lightning. This information is displayed in a red frame.”. In contrast, participants in the separation-prompt condition received the following prompt: “Please note that there will be several labelled photographs of the consequences of lightning strikes in the learning material. Among them you will find information about the formation of lightning. The information about the consequences of lightning strikes and the information about the formation of lightning are thematically different. They should therefore be treated separately. The information on the formation of lightning is displayed in a red frame.”. Base passages in conditions with instructional prompts were outlined by a red frame. An excerpt of the learning material is shown in Fig. 1. For an overview of the instructions used across the conditions, see Table 1.

**Table 1:** Overview of the instructions that were used across all four experimental conditions

Condition		Instruction			
Control & No-prompt condition		The following learning unit consists of a page about lightning and its formation. Read the content carefully. You will be asked questions about the content later. Please do not take notes while reading. You have a total time of 6.5 minutes.			
Irrelevance-prompt condition		The following learning unit consists of a page about lightning and its formation. Read the content carefully. You will be asked questions about the content later. Please do not take notes while reading. You have a total time of 6.5 minutes. Please note that there will be several labelled photographs depicting consequences of lightning strikes in the learning material. The information on these photographs is not relevant to the learning objective and will not be asked later. Relevant for the learning objective is only the information about the formation of lightning. These are located in a red frame.			
Separation-prompt condition		The following learning unit consists of a page about lightning and its formation. Read the content carefully. You will be asked questions about the content later. Please do not take notes while reading. You have a total time of 6.5 minutes. Please note that there will be several labelled photographs depicting the consequences of lightning strikes in the learning material. Below them you will find information about the formation of lightning. The information about the consequences of lightning strikes and the information about the formation of lightning are thematically different. Therefore, they should be treated separately. The information about the formation of lightning is located in a red frame.			

### Measures

For assessment of reliability, we used McDonald’s omega (ω) rather than Cronbach’s alpha because omega is less prone to error due to its core assumptions being less restrictive (for an overview see e.g. Dunn et al., 2014). R software (V4.1.2) was used to calculate McDonald’s omega (R Core Team, 2021| Psych package; Revelle 2017).

#### Prior knowledge

Prior knowledge was assessed via seven self-report items adopted from Eitel et al. (2019) based on a scale introduced by Harp and Mayer (1998). The scale comprised six weather-related statements (e.g., “I can differentiate between Cumulus- and Nimbus-clouds.“). Participants should indicate whether these statements applied to them or not. Additionally, they were asked to assess their overall knowledge in meteorology on a 5-point Likert scale (from 1 *very low* to 5 *very high*). The prior knowledge score consisted of the number of checked prior knowledge items added on the overall self-rating of prior knowledge. Thus, a prior knowledge score between 1 and 11 points was achievable (McDonald’s omega: ω = 0.74). Following the rationale of Harp and Mayer (1998), participants with a prior knowledge score of eight or higher were considered as possessing high prior knowledge (*n* = 5) and were thus excluded from further analyses.

#### Learning outcomes

Learning outcomes were assessed via an open recall sheet for recall and a multiple-choice questionnaire targeting transfer. Both scales were adapted from Eitel et al. (2019).

*Recall performance* was measured via an open question. Participants were asked to write down everything they had learned about the formation of lightning in six minutes. They were precisely instructed to report in detail and to use the knowledge acquired in the previous learning unit. This format corresponds to the recall task from Eitel et al. (2019). The open responses were scored by a rater who was blind to the assigned experimental condition. The scoring was based on a pre-existing scoring scheme from Eitel et al. (2019), based on scoring in Harp and Mayer (1998). This scoring scheme awarded points corresponding to the learning session’s ten main idea units: (1) “lightning can be defined as the difference in the electrical charge between the cloud and the ground”, (2) “warm moist air rises”, (3) “water vapor condenses and forms a cloud”, (4) “(big) raindrops and ice crystals fall, (5) “opposing airflows in the cloud cause an electrical charge”, (6) “negatively charged particles fall to the bottom of the cloud”, (7) “two leaders meet”, (8) “negatively charged particles rush from the cloud to the ground”, (9) “positively charged particles rush from the ground upwards along the same path”. (10) “the descent of (large) raindrops and ice crystals causes downdrafts”. The scoring for idea unit one was removed from the scale because of low fit with the remaining scale for internal consistency purposes. Participants received 1 point for correctly reproducing each of the idea units, 0.5 points for partially reproducing it or 0 points for failing to reproduce them correctly. Accordingly, a score between 0 and 9 points was achievable (McDonald’s omega: ω = 0.70). For observation of interrater-reliability, a second rater scored a subset of 55 answers. A high interrater reliability was obtained (Two-way random effects, accurate match, ICC = 0.89).

To measure *transfer performance*, we used a scale containing nine multiple-choice items with a sum of 41 answer options from Eitel et al. (2019). These items are based on materials used by Schmidt-Weigand and Scheiter (2011) and open tasks originally introduced by Harp and Mayer (1998). Correctly responding to the items required an inference to be drawn based on the learned information. Four of the nine items had four answer options to choose from while the remaining five items had five answer options to choose from. Participants were informed that more than one answer could be correct for each question, but were not told the number of correct answers per question. Participants scored one point for each correct answer and zero points for incorrect ones. Thus, a score between 0 and 41 points was obtainable. McDonald’s omega for the scale, assessed based on the pool of all answer options, was ω = 0.56.

#### Process variables

We assessed participants’ *perceived relevance* of seductive details, *integration avoidance* between seductive details and pertinent contents, and *relative processing time* on seductive details as learning process variables. Perceived relevance and integration avoidance were assessed via items on a 5-point Likert scale (from 1 *not at all* to 5 *very much*). They were only shown to participants in conditions including seductive details. Two items were used to assess participants’ *perceived relevance*. One item asked if participants thought that seductive details were relevant for learning and a second item asked if they thought that these would be part of the post-test, *r* = .38, *p* < .001. For assessment of *integration avoidance*, one item asked whether participants had processed seductive details and relevant material separately (“In the learning task, I tried to memorize information about formation and consequences of lightning separately. “). Participants should also indicate the percentage of time they spent processing the seductive details (“consequences of lightning strikes”) and the pertinent contents (“formation of lightning”). We calculated the *relative processing time* on seductive details by dividing the processing time on seductive details by the overall processing time (on seductive details + pertinent contents).

#### Cognitive load

Cognitive load was assessed via five items. Participants here had to state their level of agreement to different statements, answered on a 5-point Likert scale (from 1 *not at all* to 5 *very much*). The items translated to German by Eitel et al. (2019) based on Klepsch, Schmitz und Seufert (2017). Two of the items (“In the learning task, you had to process many things in your head at the same time” and “The learning task was very complex.”) referred to intrinsic cognitive load (ICL), *r* = .36, *p* < .001. The three remaining items (“In the learning task, it was difficult to recognize the most important information.”, “The presentation of the learning task was inconvenient to really learn something.” and “In this task, it is difficult to connect the key content.”) assessed extraneous cognitive load (ECL), ω = 0.75.

#### Further measures

The following measures were assessed as control variables: reading ability and visuo-spatial abilities. Conscientiousness was assessed for exploratory reasons but was not included in the analyses.

Participants’ *visuo-spatial ability* was measured via the first ten items of the Paper Folding Test (Ekstrom, 1976), which were adapted for online use. Participants had to choose between five possible pictures, the one that represents a previously folded square paper with a punched in hole when being unfolded. Mental rotation of the depicted paper was necessary to answer the tasks. Correct answers were scored one point while one point was subtracted for false answers, resulting in a possible score between − 10 and 10 points (McDonald’s omega: ω = 0.72).

*Reading ability* was assessed as possible moderating variable via the *Reading-Speed- and Comprehension-Test for Grades 5 to 12+* (LGVT 5–12+; Schneider et al., 2017) The test is used for assessment of reading competence in grades five to twelve. The test was in this case used with undergraduates because Schneider states a lack of further growth in reading abilities after grade ten. The test measures reading ability in the subscales *reading speed*, *reading comprehension* and *reading accuracy* and was adapted for the online-assessment. Out of three possible texts from the LGVT 5–12 + the text *Footboy* was chosen (2057 words). Participants should read this text as far as possible in six minutes, with the remaining reading time visible at the top of the page. While reading, participants should fill in the 47 gaps via three options from a drop-down-menu (e.g. “The footboy got a sack of food, then mounted on the [ram goat, black horse, grey horse] and rode out into the vast world”). Correct answers required knowledge about previous context of the story. After six minutes, participants were automatically redirected to the next page. There they were instructed to indicate how far they had read the text within the time limit. By clicking on a word in the text, the reading progress was shown beneath the text. Participants should transfer this information to an open text field. *Reading speed* was assessed by amount of read words. *Reading comprehension* was calculated by doubling the number of correctly filled gaps and subtracting the number of incorrect answers. *Reading accuracy* was calculated by dividing the number of correctly filled gaps and all answered gaps. This division was then multiplied by 100 and rounded off to the next whole number.

To assess participants’ *conscientiousness*, we used the subscale *conscientiousness* from the *Psychomeda Big-Five-Personality test (B5T*; Satow 2020). The scale is comprised of ten statements (e.g., “Even small slip-ups bother me”). Participants can state their agreement on a 5-point Likert scale (from 1 *Does not apply at all* to 4 *Applies exactly*), allowing for scores between 0 and 40 (ω = 0.78).

#### Control for faithful participation

All participants were asked three dichotomous control questions at the end of the survey: “Did you follow the work instruction when completing the tasks?”, “Were you able to complete the questionnaire without distraction?” and “Did you use external resources (e.g., books, Wikipedia, etc.) to complete the learning assessment?”. Furthermore, to account for technical issues, we asked participants about the type of device they used for the learning experiment (smartphone, tablet or computer). These items allowed for an assessment of the quality of collected data. Participants who did not follow the work instruction or reported to have used external resources during the experiment were excluded from further analysis. As a result, nine participants were excluded from data analysis.

### Procedure

Due to the current COVID-19 pandemic, the experiment was conducted in an online-setting, created via Questback by Unipark. After they had given their informed consent, participants answered demographic questions, and provided information about their prior knowledge and major / occupation. This was followed by working on the Paper Folding Test (three minutes). Then, participants completed the reading comprehension test (ten minutes). Next the learning phase started. In the instruction before the learning phase, all participants were informed that they would have six and a half minutes for learning the contents on the following page. Moreover, participants in the irrelevance-prompt condition were instructed that seductive details were not relevant to the learning objective. Participants in the separation-prompt condition were instructed to separately process seductive details and pertinent contents. After the learning phase, participants received questions about cognitive load, perceived relevance of seductive details (only in conditions with seductive details), their efforts to integrate seductive details and pertinent contents and use of learning time. Then, they worked on an open recall task (six minutes). Afterwards, participants worked on a transfer test, followed by the B5T subscale for conscientiousness and lastly, were asked to fill in the control questions for faithful participation. Based on a pretest (*n* = 5) the length of one session was estimated to be 60 min. Participants were fully debriefed and thanked for their participation.

## Results

Our analysis section is divided in two parts. In the first part (preliminary analyses), we applied non-parametric tests to assess whether relevant participant traits were equally distributed among the experimental conditions. Additionally, a stepwise regression analysis was conducted to identify possible relevant covariates for further analyses. The second part (hypotheses testing) comprised both a univariate ANCOVA to test for the seductive details effect and a combination of orthogonal contrast analyses and mediation analyses to test for the assumed effects of the instructional prompts on learning outcomes. An overview of the descriptive variables is depicted in Table 2.

**Table 2:** Means (and standard deviations) of main variables across all experimental conditions

	Control (n = 60)		Irrelevance-prompt (n = 66)		Separation-prompt (n = 64)		No-prompt (n = 58)		All participants (n = 248)	
Variable	*M*	*SD*	*M*	*SD*	*M*	*SD*	*M*	*SD*	*M*	*SD*
Age (in years)	22.57	2.82	23.41	7.14	23.55	5.51	24.03	6.98	23.39	5.88
Prior knowledge (Scale: 1–11)	3.49^a^	1.57^a^	3.57^a^	1.59^a^	3.25	1.64	3.31^a^	1.41^a^	3.41^a^	1.56^a^
Visuo-spatial abilities (Scale: -10-10)	3.75	3.78	3.26	4.15	3.66	3.72	4.02	3.64	3.66	3.82
Reading comprehension (Scale: -47-94)	45.63	16.14	44.06^b^	17.21^b^	45.91	15.51	44.66	14.56	45.06^b^	15.84^b^
Reading accuracy (Scale: 0-100)	90.01	10.52	89.73^b^	10.05^b^	90.07	10.22	90.26	8.66	90.01^b^	9.85^b^
ICL^d^ (Scale: 1–5)	2.93	0.87	3.15	0.88	3.14	0.74	3.21	0.94	3.11	0.86
ECL^e^ (Scale: 1–5)	2.73	0.78	2.85	0.98	3.06	0.88	3.11	1.00	2.94	0.92
Conscientiousness (Scale: 0–40)	28.73	4.33	27.95	3.65	28.47	3.60	28.50	4.18	28.40	3.93
Recall (Scale: 0–9)	2.56	1.65	2.48	1.80	2.09	1.68	1.77	1.27	2.23	1.64
Transfer (Scale:0–41)	27.30	2.98	26.39	3.53	27.20	3.27	26.38	3.19	26.82	3.27
Perceived relevance of seductive details (Scale: 1–5)	-	-	2.61	1.10	3.28	0.97	3.49	0.71	3.11^c^	1.02^c^
Integration avoidance (Scale: 1–5)	-	-	2.80	1.19	3.36	1.15	2.47	1.11	2.89^c^	1.20^c^
Relative time for processing seductive details (Percent)	-	-	0.17	0.17	0.33	0.21	0.22	0.20	0.24	0.20

*Note*:

a: N = 239 | n(control) = 59 | n (separation-prompt) = 61 | n (no prompt) = 55

b: N = 247 | n (separation-prompt) = 65 | c: N = 188

d: Intrinsic cognitive load (ICL) | e: Extranous cognitive load (ECL)

### Preliminary analyses

Kruskal-Wallis tests were used to test for equal distribution of participants’ entry variables such as age, prior knowledge, visuo-spatial abilities, reading comprehension and reading accuracy, because these variables were not normally distributed. The tests showed no significant differences between participants in the experimental conditions concerning their age, *H*(3) = 2.91, *p* = .406, prior knowledge, *H*(3) = 2.16, *p* = .540, conscientiousness, *H*(3) = 1.21, *p* = .750, visuo-spatial abilities, *H*(3) = 0.83, *p* = .841, reading comprehension, *H*(3) = 0.88, *p* = .832, and reading accuracy, *H*(3) = 0.63, *p* = .890. A χ^*2*^-test revealed that participants’ gender was also equally distributed among the experimental conditions, χ^*2*^(3) = 2.60, *p* = .457. Most of these entry variables correlated with at least one of the two main dependent variables of recall and transfer performance. Therefore, they were entered into stepwise linear regression models, separately for recall and transfer, to narrow down which of the entry variables explained the most unique variance in the dependent variables, and thus qualifies as covariate for hypothesis testing. The inclusion criterion was based on *p*-values with a threshold of 0.10. For recall, the two entry variables of reading comprehension (*b* = 0.019, *SE* = 0.007, *β* = 0.176, *p* = .004) and visuo-spatial ability (*b* = 0.130, *SE* = 0.026, β = 0.304, *p* < .001) met the inclusion criterion, whereas the two entry variables of reading comprehension (*b* = 0.043, *SE* = 0.013, β = 0.204, *p* = .001) and prior knowledge (*b* = 0.364, *SE* = 0.130, β = 0.176 *p* = .006) met the inclusion criterion for transfer.

### Seductive details hypothesis

**Fig. 2:**
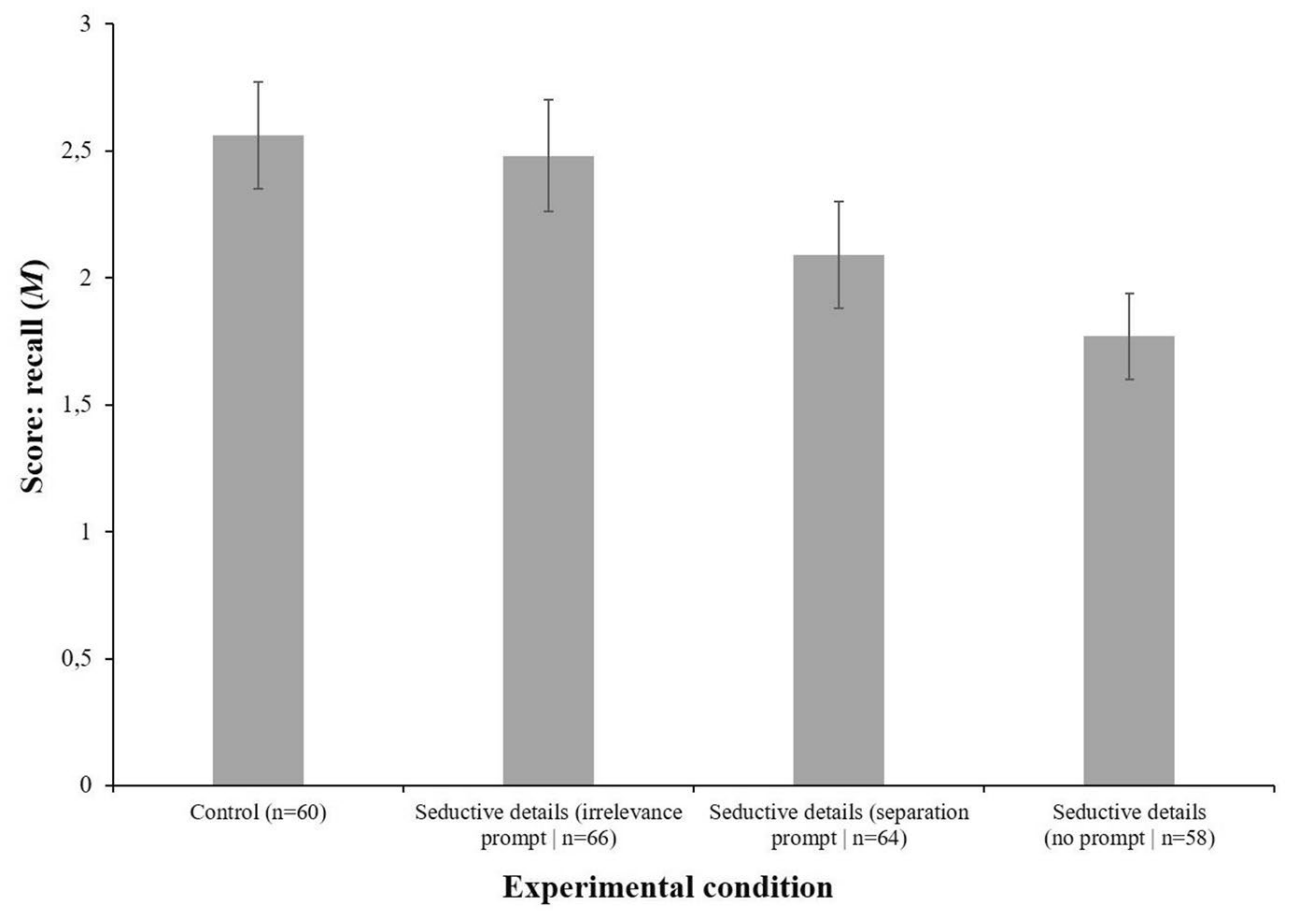
Results for recall performance (mean) across different all experimental conditions (Recall Scale: 1–5). Error bars represent standard errors of mean

To test for the seductive details effect, we conducted two analyses of covariance (ANCOVA) with recall and transfer score as dependent variables, the presence of seductive details as factor (control condition vs. no-prompt condition). Reading comprehension and visuo-spatial abilities were used as covariates for recall and reading accuracy as well as prior knowledge as covariates for transfer. Descriptive values are shown in Table 2. Our analyses showed a significant difference for recall, *F*(1,112) = 16.430, *p* < .001, η_*p*_^*2*^ = 0.074, but not for transfer, *F*(3,108) = 2.19, *p* = .142, η_*p*_^*2*^ = 0.020. The post-hoc power analysis for recall revealed a power of 0.85 for the analysis. For recall, participants in the control group significantly outperformed participants in the no-prompt condition, revealing a seductive details effect (see Fig. 2). Thus, only the results for recall performance supported the seductive details hypothesis in our study.

### Diversion hypothesis

For the diversion hypothesis to be true, seductive details should hamper learning only when they are perceived as relevant so that students form mental models around them, rather than around the pertinent contents. Thus, students in the irrelevance-prompt condition (+ 1) should perform similarly well as students in the control condition (+ 1), and the both of them better than students in the no-prompt condition (-2), because only the latter should show diversion. We entered this focal contrast (+ 1, + 1, -2) in an ANCOVA together with a residual contrast that compared performance in the control condition to performance in the irrelevance-prompt condition (contrast code: +1, -1, 0). We entered recall and transfer, separately, as dependent variables, and reading comprehension and visuo-spatial abilities as covariates in the analysis. This analysis revealed the focal contrast (+ 1, + 1, -2) to reach significance for recall, *F*(1,177) = 11.580, *p* = .001, η_*p*_^*2*^ = 0.061, but not for transfer, *F*(1,168) = 0.907, *p* = .342, η_*p*_^*2*^ = 0.01. A post-hoc power analysis for recall determined a power of 0.93. The focal contrast thus revealed that participants in the control condition and the irrelevance-prompt condition outperformed participants in the no-prompt condition regarding recall but not transfer. The residual contrast (+ 1, -1, 0) did not reach significance for either recall, *F*(1,177) = 0.015, *p* = .901, η_*p*_^*2*^ < 0.001, nor transfer, *F*(1,168) = 1.690, *p* = .195, η_*p*_^*2*^ = 0.010.

**Fig. 3:**
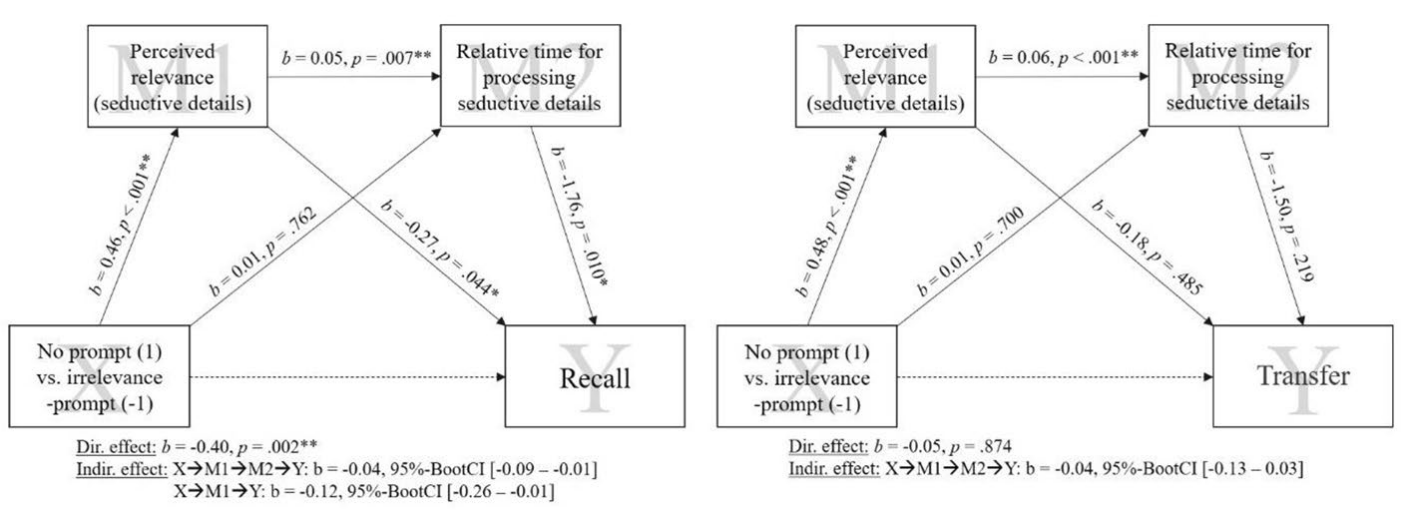
Results of the two-step mediation analysis models representing the diversion hypothesis with recall (left) and transfer (right) as dependent variables

These findings (partially) suggest the diversion hypothesis to be true, because seductive details hampered (recall) performance only when they were perceived as relevant, and mental models were presumably constructed around them rather than around pertinent contents. To test diversion more directly, we tested the following mediation models for recall and transfer: Seductive details without prompts should hamper learning outcomes (compared to receiving irrelevance prompts) via higher perceived relevance of seductive details which should lead to an increase in the time spent on processing seductive details compared to pertinent learning contents (as an approximation to mental model formation). The analysed mediation models are depicted in Fig. 3. For recall, this mediation model was significant (*b* = -0.04, 95% BootCI [-0.09, -0.01]), supporting the diversion hypothesis (see also Fig. 3). Also, a one-step mediation via perceived relevance only reached significance for recall (b = -0.12, 95%BootCI [-0.26, -0.01]). For transfer, the mediation model did not reach significance (*b* = -0.01, 95%BootCI [-0.03, + 0.01]). For all models, bootstrap confidence intervals are based on a bootstrap sample of n = 5000. For recall, reading comprehension and visuo-spatial abilities were entered as covariates. For transfer, reading comprehension and prior knowledge were entered as covariates.

### Disruption hypothesis

For the disruption hypothesis to be true, seductive details should hamper learning when students try to integrate and establish coherence between the details and the pertinent contents. Therefore, students who receive a separation prompt about the details referring to a separate topic should avoid integration efforts and thus perform better than students who learn with seductive details without prompt. For recall, students who received the separation prompt did not outperform students in the no-prompt condition, not supporting the disruption hypothesis, *F*(1,113) = 4.49, *p* = .106, η_*p*_^*2*^ = 0.023. For this analysis, a post-hoc power analysis determined a power of 0.37. Our analysis on transfer also revealed no significant difference between both groups, *F*(1,110) = 1.16, *p* = .283, η_*p*_^*2*^ = 0.010.

**Fig. 4:**
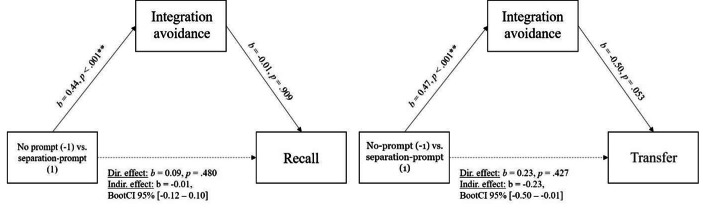
Results of the one-step mediation analysis models representing the disruption hypothesis with recall (left) and transfer (right) as dependent variables

To test disruption more directly, we analysed in a mediation model whether the separation prompt would foster learning outcomes (recall and transfer) compared to no prompt by increasing integration avoidance. The analysed mediation models are depicted in Fig. 4. The analyses revealed no significant mediation via integration avoidance for recall (*b* = -0.01, 95%BootCI [-0.12, + 0.10]), but a significant mediation for transfer (*b* = -0.23, 95%BootCI [-0.51, -0.01]). In both analyses, the contrast between the separation-prompt condition and the no-prompt condition influenced participants integration avoidance positively (recall: *b* = 0.44, *p* < .001; transfer: *b* = 0.47, *p* < .001) which, in turn, did not influence recall (*b* = -0.01, *p* = .909), but transfer just missed significance (*b* = -0.50, *p* = .053). This mediation, against our hypothesis, suggest that students in the separation-prompt condition avoided integration of seductive details with pertinent contents to a stronger degree, which led to worse transfer performance. These findings are not reflected in the contrast analyses.

### Cognitive load hypothesis

**Fig. 5:**
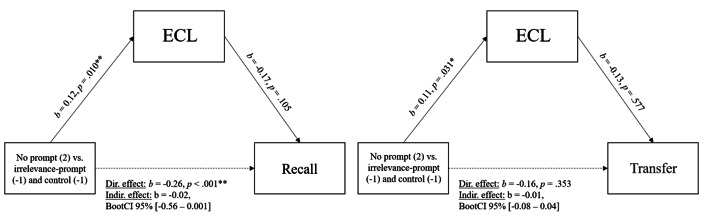
Results of the one-step mediation analysis models representing the cognitive load with recall (left) and transfer (right) as dependent variables

For the cognitive load hypothesis to be true, seductive details should hamper learning when they increase ECL. Specifically, students in the no-prompt condition (-2) should show higher ECL and, in turn, worse recall and transfer than students in control condition (+ 1) and in the irrelevance-prompt condition (+ 1). Our analyses showed no significant mediation via ECL for recall (*b* = -0.02, 95%BootCI [-0.56–0.001]) and transfer (*b* = 0.11, 95%BootCI [-0.08–0.04]). For both analyses, the contrast between no-prompt condition and irrelevance-prompt as well as control condition significantly affected ECL (recall: *b* = 0.12, *p* = .010; transfer: *b* = 0.11, *p* = .031). The amount of experienced ECL, however, did not influence recall (*b* = -0.17, *p* = .105) and transfer (b  =  -0.13, p = .577) significantly (see Fig. 5).

## Discussion

In this study, we tested whether diversion or disruption mainly drives the seductive details effect. We compared effects on learning processes and outcomes from two instructional prompts (irrelevance, separation) designed to either reduce the seductive details’ diverting or disrupting potential. In a nutshell, results revealed evidence in favor of diversion, and no evidence for disruption to mainly drive the seductive details effect.

### Diversion drives the seductive details effect

According to our diversion hypothesis, seductive details are detrimental to learning (a) when students form mental models around irrelevant contents included in the seductive details rather than around the pertinent ones (see also Harp & Mayer 1998). (b) This should only be the case, when students erroneously consider the seductive details as instructionally relevant. We found evidence to support both parts of this hypothesis. Concerning (a), we found that students who attended relatively longer to seductive details had worse learning outcomes for recall (potentially reflecting mental model construction). Concerning (b), prompting students about the irrelevance of seductive details reduced students’ perceived relevance and relative processing time for seductive details, which in turn fostered recall performance. In fact, prompting students about the irrelevance of seductive details made the seductive details effect disappear for recall, albeit there were no effects of seductive details on transfer in the present study. This partly supports the idea of diversion being the main driver of the seductive details effect.

Our results are in line with previous findings from Bender et al. (2021b) and Eitel et al. (2019), who also found irrelevance prompts to make the seductive details effect disappear. Compared to previous research, the present study obtained these results in an online learning situation. This learning context might require more self-control while learning, which speaks for the robustness of both the seductive details effect and its diversion explanation for recall, but not for transfer. Furthermore, the present results align well with findings from other studies that support the diversion explanation using various methodological approaches (Bender et al., 2021; Harp & Mayer, 1998; Chang & Choi, 2014).

### Disruption does not drive the seductive details effect

According to our disruption hypothesis, seductive details are detrimental to learning when students (unsuccessfully) try to integrate them with the pertinent contents in an effort to establish coherence. Prompting students about seductive details referring to a different topic (separation prompt) should avoid such integration efforts, and thus prevent from negative effects on learning outcomes. We found no evidence supporting this hypothesis. Students who received the separation prompt reported reduced effort to integrate seductive details with pertinent contents. However, receiving the separation prompt did not make the seductive details effect disappear. Rather, reporting higher integration avoidance (due to the separation prompt) did not foster but hamper learning outcomes, as reflected in the negative correlation between integration avoidance and transfer performance. From this pattern of results, we conclude that it is unlikely that disruption did drive seductive details effect. This is somewhat in line with the meta-finding of few previous studies showing (only indirect) support for the disruption hypothesis (Wade et al., 1993; Lehman et al., 2007), while others studies did not show a meaningful impact of disruption on learning outcomes (Harp & Mayer, 1998; Bender et al., 2021). This study supports and extends previous research by providing no evidence in favor of the disruption hypothesis.

### Limitations and future research

Note that the beneficial effects of the irrelevance prompt on learning performance may not unequivocally speak in favor of diversion as the main driving force behind seductive details, because the irrelevance prompt may have also reduced the disruption process. However, there was no significant difference for reported integration avoidance between the irrelevance-prompt group (M = 2.80, SD = 1.19) and the no-prompt group (M = 2.47, SD = 1.11) suggesting that preventing from disruption was not the main process to explain why the irrelevance prompt was effective for learning. Moreover, we found no significant beneficial effects of the separation prompt on learning performance. Thus, we interpret our findings as evidence in favor of diversion and in disfavor of disruption. Nevertheless, additionally obtaining eye tracking data could provide even more insights into the process of integration (avoidance) and be further helpful to distinguish between the disruption and diversion explanation.

We were not able to replicate the finding that extraneous cognitive load acts as a mediator for the detrimental effects of seductive details as previously found by Eitel et al. (2019). This finding is consistent with other learning studies depicting favorable effects for instructional designs that evoke higher ECL (for an overview see Skulmowski & Xu 2022). One possible reason for our finding could be that we carried out our study in an online setting, where less control is possible than in a lab setting. This may reduce the obtained effect sizes. It is unclear whether this could also be the case for effects caused by ECL. However, by having a high number of participants per experimental condition (around 60), we still managed to generally find significant effects as previous lab research did (e.g., Eitel et al., 2019). Moreover, we tried to obtain high data quality by asking students at the end of the experiment whether they faithfully participated; we discarded their data if they disagreed. We believe that the participants were honest about this, since answering this question yielded no negative consequences for them. An advantage of the online setting is that this setting is representative for many learning situations since the outburst of the COVID-19 pandemic. There is thus a certain ecological validity to our results. Nevertheless, further research may switch the research setting to test for the robustness of the present findings. Against our hypothesis, our data did not show a seductive details effect for transfer. We suspect this to be the case because of low internal consistency of the multiple-choice transfer test (ω = 0.56). Consequently, no definitive conclusions about the potential effects of seductive details on comprehension can be drawn from the available data. We used this transfer test to keep our design close to the one from Eitel et al. (2019). Here, the same test was used and reached a similar internal consistency (Cronbach’s α = 0.42). The use of an open transfer test instead of a multiple-choice test in future studies might reveal effects for transfer performance.

With regard to our learning material, it is true that the control group differed from the other three groups regarding the amount of information to be processed. In contrast to the other groups, learners in the control group received less learning content, and consequently had less information to process in the same amount of time, which may have had a beneficial impact on their learning outcomes. Yet, prior studies did not indicate effects of time-pressure due to the higher amount of learnt information on learning performance (see Eitel et al., 2019). Moreover, the experimental design we used in our study (learning material vs. learning material + SD) is commonly used in studies researching seductive details (e.g. Harp & Mayer 1998), which is why we also did not test whether seductive details would be recalled. Using such tests in future studies may provide further insights into what precisely students learn when receiving materials containing seductive details. By mimicking Harp and Mayer’s (1998) design, we kept our design and by extension our results comparable to other studies in this field of research.

It is further to note that we obtained process data indirectly via self-reports. For instance, we had a two-item scale to measure processing times on the different parts on the page (seductive details, pertinent contents). It is possible that these self-reports contain individual biases. For future research, we therefore recommend additionally assessing objective process data, for instance by means of eye tracking. Such data could provide new insights into the cognitive processing of students and help to validate the present self-reports on integration avoidance by relating them to eye-gaze transitions between pertinent contents and seductive details (see e.g., Korbach et al., 2016). Regarding the separation prompt, we assessed participants’ avoidance to integrate seductive details with pertinent content with a single item. We consider the single-item measurement adequate here, because the underlying construct is not multidimensional, and easy to narrow down (see Allen et al., 2022). In future studies, however, it might be worthwhile investigating whether more elaborate measurements (e.g., process data) of this construct would provide additional insights.

### Theoretical and practical implications

The present results suggest diversion, and not disruption, to drive the seductive details effect. Students who thought the details were relevant reported longer processing times, and in turn had worse learning outcomes. On a theoretical level, this supports the idea of seductive details being detrimental when they stimulate students to think more deeply about the seductive details, or contents related to the details, but not about the pertinent contents (see also Bender et al., 2021b). Seductive details hamper learning when they make students activate and integrate prior knowledge that is inadequate to solve the (learning) task at hand. This finding underscores the importance of (correct) prior knowledge retrieval for successful learning as also conceptualized within the cognitive theory of multimedia learning (Mayer, 2014). In other words, adding seductive details is detrimental when the added seductive information is deeply processed. As seductive details are interesting and irrelevant, they are often processed deep enough to exert their detrimental effects compared to not presenting them (see Rey 2012; Sundararajan & Adesope, 2020) or compared to being prompted that they are irrelevant and can be ignored (Eitel et al., 2019). Based on the present pattern of results, we can conclude that deeper processing of seductive details, whether integrative or not, is detrimental.

The present results also suggest that students who received irrelevance prompts were unaffected by seductive details even though they reported having tried to establish some coherence between seductive details and pertinent contents. It seems that processing the details on a more superficial level did not hamper learning, even though it likely made initial coherence formation a bit more difficult. Knowing about the details’ irrelevance seems to have made students compensate for superficial processing difficulties due to seductive details by still engaging in sufficient deep-level processing of pertinent contents (e.g. on the situation model level; van Dijk & Kintsch 1983). The latter is, however, still a hypothesis and further research may test it by instructing students to engage in deep-level processing of pertinent contents in the presence or absence of seductive details.

From a practical point of view, the present results suggest that seductive details have detrimental effects also in online learning sessions with reduced external control. The safest way to prevent from harmful effects of seductive details is to omit them from lesson materials or e-book pages entirely. As this is not always possible, telling students about the irrelevance of the seductive details for the present task is a small but promising intervention. More specifically, a teacher may tell students “this part is just for fun”, or “watch here to loosen up when you need a short break, but then return to the pertinent contents displayed here”. Instructional prompts have the potential to reduce the amount of processed irrelevant information and thus enhance learning performance. On a more general note, informing teachers and instructional designers about the diverting potential and detrimental effects of seductive details in (online) study materials, as found in this study as well as in previous research (see e.g., Bender et al., 2021b), seems promising to reduce detrimental effects of seductive details in educational practice.

## Data Availability

Available data can be provided upon request to the corresponding author.

## Footnotes

1 For learning with text, the construction-integration model (Kintsch, 1998) could alternatively be used to address the disruption hypothesis. According to this model, adding conceptually unrelated information to a text interrupts its logical structure as well as text processing based on this structure.
